# Bone Matrix Non-Collagenous Proteins in Tissue Engineering: Creating New Bone by Mimicking the Extracellular Matrix

**DOI:** 10.3390/polym13071095

**Published:** 2021-03-30

**Authors:** Marta S. Carvalho, Joaquim M. S. Cabral, Cláudia L. da Silva, Deepak Vashishth

**Affiliations:** 1 Center for Biotechnology and Interdisciplinary Studies, Department of Biomedical Engineering, Rensselaer Polytechnic Institute, Troy, NY 12180, USA; 2 Department of Bioengineering and iBB—Institute for Bioengineering and Biosciences, Instituto Superior Técnico, Universidade de Lisboa, 1049-001 Lisboa, Portugal; joaquim.cabral@tecnico.ulisboa.pt (J.M.S.C.); claudia_lobato@tecnico.ulisboa.pt (C.L.d.S.); 3 Associate Laboratory i4HB—Institute for Health and Bioeconomy, Instituto Superior Técnico, Universidade de Lisboa, 1049-001 Lisboa, Portugal

**Keywords:** non-collagenous proteins, extracellular matrix, bone tissue engineering, biomimetic scaffolds

## Abstract

Engineering biomaterials that mimic the extracellular matrix (ECM) of bone is of significant importance since most of the outstanding properties of the bone are due to matrix constitution. Bone ECM is composed of a mineral part comprising hydroxyapatite and of an organic part of primarily collagen with the rest consisting on non-collagenous proteins. Collagen has already been described as critical for bone tissue regeneration; however, little is known about the potential effect of non-collagenous proteins on osteogenic differentiation, even though these proteins were identified some decades ago. Aiming to engineer new bone tissue, peptide-incorporated biomimetic materials have been developed, presenting improved biomaterial performance. These promising results led to ongoing research focused on incorporating non-collagenous proteins from bone matrix to enhance the properties of the scaffolds namely in what concerns cell migration, proliferation, and differentiation, with the ultimate goal of designing novel strategies that mimic the native bone ECM for bone tissue engineering applications. Overall, this review will provide an overview of the several non-collagenous proteins present in bone ECM, their functionality and their recent applications in the bone tissue (including dental) engineering field.

## 1. Introduction

**Figure 1:**
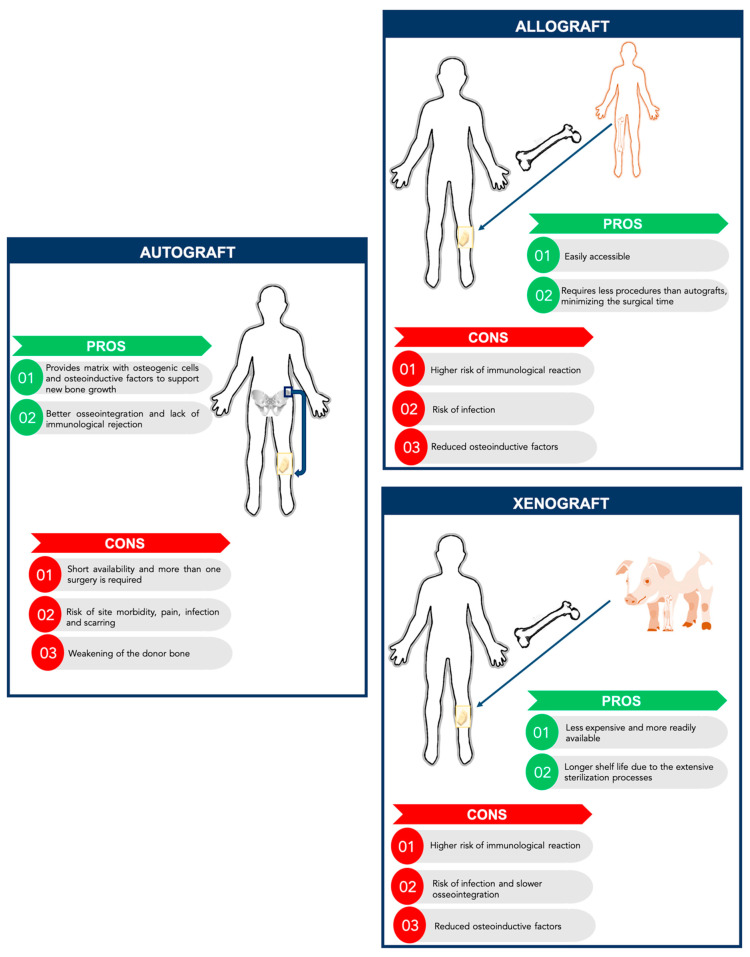
Comparison between autografts, allografts, and xenografts: Advantages and disadvantages.

New promising solutions for bone tissue engineering have been developed over the last years following the dramatic increase of the number of bone-related medical conditions that require clinical interventions. In fact, each year, more than one million non-union fractures are treated in the United States [1]. Moreover, 5–10% of the bone fractures that occur worldwide do not heal [1]. Besides bone fractures, bone tissue can also be damaged by traumas, tumors, infections, or bone diseases. Furthermore, new strategies to engineer bone tissue are required as an alternative to the use of bone grafts, addressing the increasing worldwide incidence of bone disorders in an aging society severely impacted by obesity, lack of exercise, and with reducing healing capacity [2]. Even though bone tissue engineering appears as a promising alternative, to date the gold-standard treatment for bone regeneration still relies on bone grafts, autologous, allogenic, and xenogeneic grafts [1] (Figure 1). These approaches have some limitations and are not ideal for bone regeneration. Autografts have been applied since they can provide a matrix with osteogenic cells and osteoinductive factors to support new bone growth, without having immunological rejection and promote better osseointegration. Nonetheless, the availability of quality graft material is limited and possible complications may occur, such as pain, infections, scarring, and weakening of the donor bone. Moreover, there is a high morbidity associated with this procedure, since more than one surgery is needed [1,3]. Allografts, usually harvested from cadavers, have also some limitations, namely the higher risk of immunologic rejection and infection though it requires less procedures than autografts, minimizing the surgical time and accelerating the patient recovery [3]. As an alternative to allografts, xenografts consist of transplantation of bone tissue across species. The most common xenograft used in orthopedic surgery is bovine derived. Xenografts have some advantages compared to other grafts, such as being readily available due to the abundance of donor bone tissue and being less expensive than allografts [2,3]. In fact, commercially-available xenografts are approximately one-tenth the price of commercially-available allografts. Also, because of the extensive sterilization processes, their shelf life is generally long. However, xenografts present several challenges such as the risk of disease transmission and a higher risk of immune response of the host tissue compared to allografts [3]. Moreover, xenografts require intensive sterile processing, which can decrease their osteoinductive properties.

**Figure 2:**
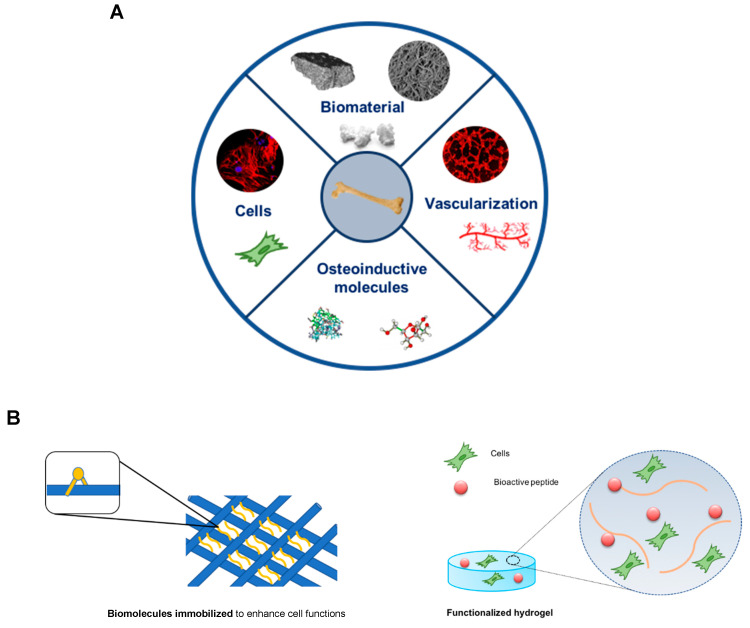
Bone tissue engineering strategies. (**A**) The bone tissue engineering paradigm highlights (1) Biomimetic scaffold, (2) osteogenic cells, (3) osteoinductive molecules, and (4) vascularization. (**B**) Schematic representation of biomolecules immobilized into a porous scaffold (left) and a functionalized hydrogel with bioactive peptides and cells incorporated (right).

Bone tissue engineering has the potential for solving these problems by combining different elements such as cells, molecules, and scaffolds. The standard tissue engineering approach uses a combination of growth factors, scaffolds and osteogenic cells (triangular concept). However, Giannoudis and colleagues developed and discussed a new concept, the diamond concept, in which a fourth element, vascularization, should be also considered as a contributor to bone healing. Thus, the diamond concept in bone tissue engineering combines four basic elements [4]: (i) A biomaterial with osteogenic ability for bone formation that acts as a scaffold for the other elements; (ii) osteogenic cells capable to creating or inducing new bone formation at the defect site; (iii) osteoinductive molecules that trigger cells and recruit resident cells to form new functional bone tissue; and (iv) vascularization to support the viability of the defect site thus allowing the diffusion of oxygen and nutrients to the defect region (Figure 2).

Several scaffolds have been developed for bone tissue engineering, including natural biomaterials (such as collagen, gelatin, and chitosan), ceramic implants (such as hydroxyapatite), polymeric synthetic materials (such as polylactic acid (PLA) and polyglycolic acid (PGA)) and composite scaffolds [5]. Even though materials science technology has resulted in clear improvements for bone regeneration, challenges to achieve functional and mechanically competent bone growth still remain. One approach in the design of bone scaffolds involves the production of biomimetic bone matrices, in which the bone replacement material should interact with the surrounding tissues by biomolecular recognitions [6]. Among the molecular signals used for bone tissue engineering applications, the number of alternatives is yet smaller compared to the number of different scaffolds materials that can be used. Bone morphogenetic proteins (BMPs), a group of growth factors, play the leading role in the field, being able to promote proliferation and differentiation of osteogenic cells [7]. BMP-derived peptides have been widely used for bone tissue engineering applications, in particular derived from BMP-2 and BMP-7 [6]. BMP-2 peptides have been shown to promote osteogenic differentiation of MSCs [7,8]. Lin and co-workers developed a copolymer membrane loaded with a novel synthetic BMP-2 derived peptide, P24, and observed enhanced osteogenic differentiation of MSCs in vitro and bone regeneration in vivo [8]. Furthermore, BMP-2 and -7 received approval from the FDA and the EMA to be used in combination with type I collagen for the treatment of severe tibial fractures and posterolateral spinal fusions [9,10,11]. Although being widely used, several side effects of BMPs have been reported, such as postoperative inflammation and associated adverse effects, ectopic bone formation, osteoclast-mediated bone resorption, and inappropriate adipogenesis [12]. BMPs have other drawbacks, such as the high costs of production and the high doses required, raising questions about their cost-effectiveness [13]. In addition to above, calcitonin gene-related peptide (CGRP) has been widely applied due to its bone regeneration potential. CGRP might play a crucial role in promoting osteoblast proliferation and differentiation by bonding with functional receptors and transporters on the osteogenic cells and by stimulating growth factors production, such as BMP-2 [14]. In fact, Mi and colleagues demonstrated that CGRP administration increased new bone formation by promoting MSCs migration and differentiation [14]. A recent study from Lai and co-workers presented a new strategy to immobilize CGRP onto TiO_2_ nanotubes through polydopamine [15]

Recently, the field has shifted towards investigating the interaction between extracellular matrix (ECM) proteins and cell membrane receptors [5]. This approach avoids the use of growth factors and better mimics the bone ECM, reducing the side effects and increasing the efficiency of bone healing process. ECM proteins can be used intact or reduced to peptides with specific sequences that will trigger the action required. Therefore, these new osteoinductive peptides are easy and less expensive to manufacture, more unlikely to elicit immune responses due to their small size and stable in physiological conditions [16]. An attractive strategy consists of combining different ECM peptides to enhance cellular processes, such as adhesion and proliferation but also to promote osteogenic differentiation and angiogenesis. Combining peptides with important functions/properties increases the effectiveness and versatility of the final product to be used in clinical applications. In spite of the great improvements on incorporating ECM peptides on biomimetic materials, the influence of non-collagenous bone ECM proteins on osteogenic differentiation remains to be evaluated. This review aims to present a summary of the different non-collagenous proteins found in bone ECM and their important functions in context of bone tissue engineering applications, specifically on their role in cell adhesion, proliferation, osteogenic differentiation, and angiogenic capacity.

## 2. Bone Extracellular Matrix: Characterization, Properties, and Quality

**Figure 3:**
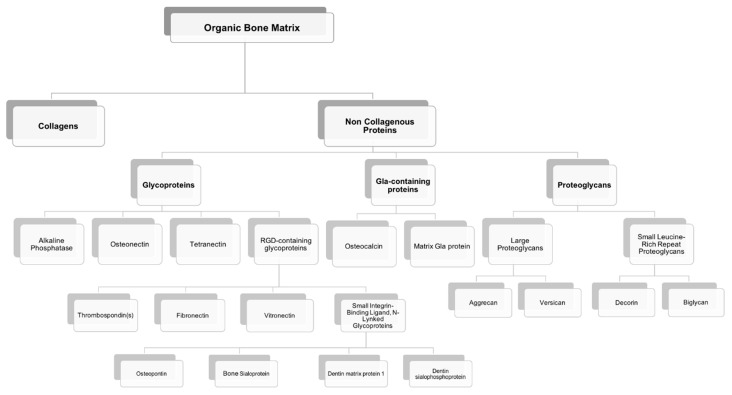
Organic components of the bone extracellular matrix.

**Figure 4:**
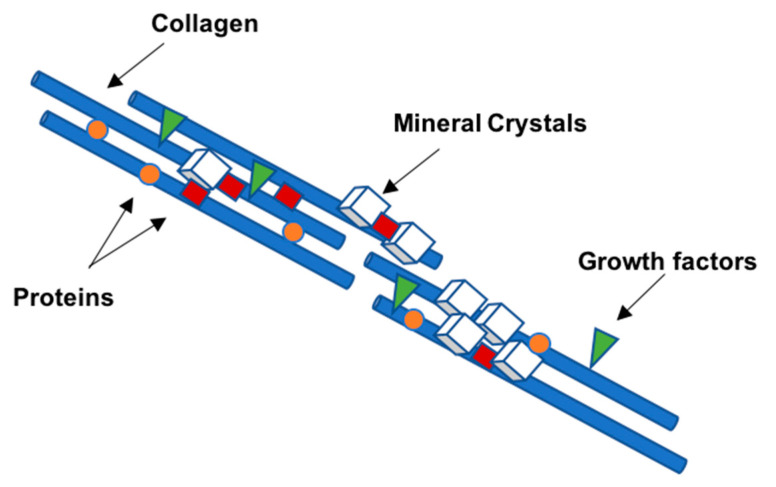
Schematic diagram representing the organization of the collagen molecules reinforced with calcium phosphate nanocrystals, proteins, and growth factors arranged in a semi-regular pattern.

Most of the outstanding properties of the bone are related to its matrix constitution [17]. Bone ECM has two components: A mineral part comprising of hydroxyapatite (70–90%) and an organic part (10–30%) composed primarily of collagen (approx. 90% of organic matrix) with the rest being non-collagenous proteins (~10%) [17,18]. The organic matrix of bone is mainly composed of collagen, however, Herring and co-workers identified the presence of other non-collagenous proteins [19] (Figure 3). Type I collagen is the most prevalent protein in the body and can be found not only in mineralized but also in non-mineralized tissues, playing a critical role in the structure and function of different skeletal tissues [18]. However, type I collagen is not the only protein involved in mineralization. Improved technologies have led to the isolation of a large number of non-collagenous matrix proteins. It is known that some matrix proteins bind to collagen forming fibrils. Thus, collagen serves as a scaffold upon which nucleators of hydroxyapatite, such as non-collagenous proteins, are present (Figure 4) [18,19,20]. Although some studies have already described the potential role of these non-collagenous proteins, their contributions and role in bone tissue engineering applications remain to be well investigated. Moreover, bone ECM quality may be determined not only by the nature of collagen type I, but also by mineral and non-collagenous proteins composition [21,22]. Using different characterization techniques and diseased mice models, it has been demonstrated that the nano-structural organization influences bone properties. In fact, several diseases related with deregulation of type I collagen and mineralization showed impairment of bone quality and other bone properties, such as bone fragility and strength. Mice with osteogenesis imperfecta, a condition derived from mutation in type I collagen, presented bone fragility and reduction in strength [23,24]. Osteopetrosis is a condition responsible for hypermineralization of bone that increases bone fragility [25] and involves altered interactions between collagen and mineral component that modify the nature of organization in bone at the nanometer scale. Non-collagenous proteins have also been suggested to influence the mechanical quality of bone matrix. Studies on osteopontin (OPN) showed that it behaves like “glue” in bone [26]. In the presence of calcium ions, OPN is capable of sacrificial bonding, a nanoscale mechanism that dissipates energy and inhibits crack growth. Osteocalcin (OC), the most abundant bone specific non-collagenous protein, complexes with OPN [27] and regulates bone mineralization through its strong affinity to hydroxyapatite. Previous works from our group found that fracture in bone initiates as dilatational bands that form as a result of OC-OPN interaction. In the absence of either protein, the complex is disrupted, resulting in a dramatic loss of toughness [28].

## 3. Non-Collagenous Bone Matrix Proteins

**Figure 5:**
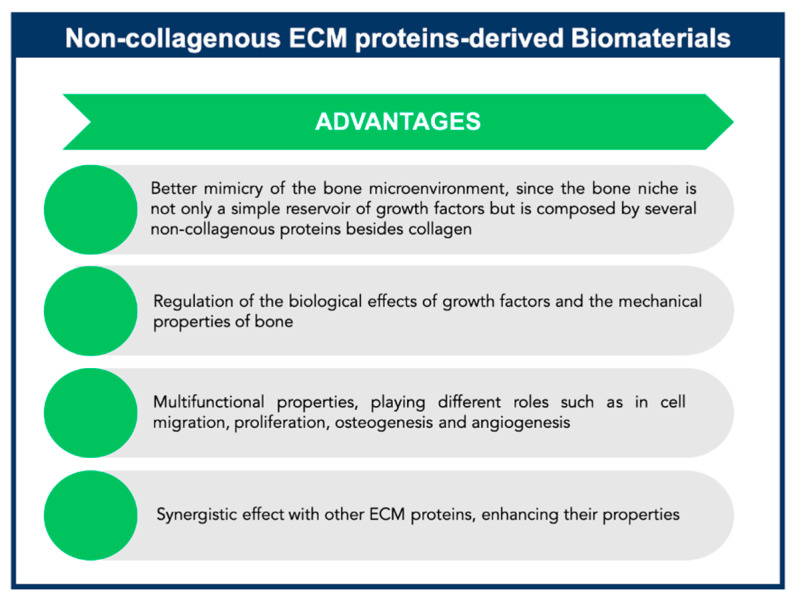
Advantages of non-collagenous extracellular matrix (ECM) proteins-derived biomaterials for Bone Tissue Engineering applications.

Non-collagenous proteins have been isolated from bone and have been found to be biologically active, even though their functions are not yet completely understood. Based on their localization patterns, each of these proteins may perform different functions. Therefore, it is extremely important to better understand the properties and functions of these proteins, aiming to design innovative strategies for bone tissue engineering applications. It has been speculated that non-collagenous proteins might have an important role in cell attachment, cell differentiation, and regulation of hydroxyapatite minerals deposition [29]. Some of these proteins may be multifunctional, playing different roles in the bone, thus defining a single function may not be sufficient. Also, some of these proteins might act together, having a synergistic effect on cellular behavior and mechanical properties of bone, or they can compensate some effects resulting from deregulation of the levels of other non-collagenous proteins present in bone matrix (Figure 5). However, non-collagenous proteins may also present some drawbacks such as limited information about their mechanism of action, high-water solubility, and limited availability.

Interestingly, not all types of bones contain the same amount of non-collagenous bone proteins. In humans, for example, cortical bone contains 30× more OC than trabecular bone, but trabecular bone contains 21× more osteonectin (ON) [30]. Moreover, it is possible to find non-collagenous proteins in some other tissues besides bone, specifically OPN and ON present a general tissue distribution. Bone sialoprotein (BSP) and OC are also found in other mineralizing tissues, such as dentin. Therefore, importance of these proteins in bone physiology cannot be underestimated. Indeed some studies have reported that mutations in some of these proteins may result in abnormal bone [29].

The multifunctional properties of these non-collagenous proteins make them attractive agents for incorporation within an appropriate scaffold to enable stem cell-based bone tissue engineering (Figure 5). These proteins can be used successfully as signaling molecules to direct stem cell recruitment, attachment, and differentiation and create a mature and mineralized extracellular matrix.

In bone, non-collagenous proteins are mainly composed of two major types: Glycoproteins and gamma-carboxyglutamic acid (Gla)-containing proteins; however some proteoglycans can also be found in smaller content [31,32]. The most relevant and abundant glycoproteins are represented by alkaline phosphatase (ALP), ON, and the Arginine-Glycine-Aspartic acid (RGD)-containing proteins, which include, but are not limited to, OPN and sialoproteins. Of the Gla-containing proteins, OC is the major component.

## 4. Exploiting Non-Collagenous Proteins in Bone Tissue Engineering Applications: An ECM Mimicking Approach

For regeneration of soft and hard tissues, several synthetic and natural materials have been used to create “ideal” scaffolds that mimic and function as bone tissue. These scaffolds should be porous, biocompatible and biodegradable in order to promote cell recruitment, migration and proliferation. Additionally, these scaffolds should also stimulate neovascularization into the graft, incorporate into the host microenvironment and form new healthy tissue [33]. Such new designs have indeed led to great breakthroughs and improvements in bone tissue engineering, however their development has also highlighted several shortcomings over the years. Although fabrication of synthetic materials can be easily reproducible, they often lack the biological cues required for engineering new tissue [33]. In contrast, scaffolds derived from natural materials, such as type I collagen, possess bioactive motifs that can guide cell adhesion, proliferation, differentiation, and tissue regeneration and have demonstrated better osteoinductive properties. However, these natural scaffolds suffer from lack of reliable and reproducible quality standards and greater batch-to-batch variation [34,35]. To combine the advantages of natural and synthetic materials and minimize the drawbacks of each, hybrid scaffolds have been designed for bone tissue engineering. Thus, researchers have been recently focused on designing and engineering materials with structure, composition and functions similar to the bone ECM. Moreover, the defined structure of biomaterials can also provide mechanical cues and modulate the microenvironment, controlling cell behavior. Thus, when fabricating new scaffolds for bone tissue engineering applications, it is important to consider the influence of physical cues, such as porosity, pore structure, roughness, and stiffness. Aiming to tune the microstructure of the biomaterial, different methods can be applied, such as 3D printing and electric field-assisted techniques, which improve scalability and control of scaffold microstructure [2,36].

The effort to functionalize synthetic scaffolds with biological cues, such as growth factors and ECM peptides, to form new tissue involves elicitation of specific cellular responses that may be absent in the native tissue (Figure 2) [37,38]. In fact, ECM can provide an adhesive substrate to which integrin and other adhesive cell receptors can bind, modulating several signaling cascades. ECM peptides can interact with cell-surface receptors, regulate signaling cascades that control cell development and determine gene expression [39]. Therefore, the properties of materials can be enhanced, specifically by promoting osteogenic differentiation and by inducing expression of osteogenic marker genes in osteoblasts [33]. Among the several signaling molecules, BMPs have been used clinically. Instead of the use of growth factors, another common strategy is the incorporation of cell-binding peptides into biomaterials, mimicking naturally occurring processes such as cell-ECM signaling, cell proliferation and differentiation [40]. The most commonly used peptide for surface modification is RGD [41,42], the signaling domain derived from fibronectin (FN) and laminin and also found in collagen. These peptides can be chemically attached to polymers to facilitate cellular interactions at an injury site. Specifically, RGD peptides have been shown to enhance proliferation, differentiation and mineralization when attached to the surface of various biodegradable materials [37,42,43]. Biomimetic PLA scaffolds modified with RGD peptides have been fabricated to promote the attachment and proliferation of osteoblasts [42]. Moreover, it has been reported that, by controlling the distribution of RGD on hydrogels by nanopatterning, it is possible to maximize its beneficial effects on adhesion, viability, and differentiation of mesenchymal stem/stromal cells (MSCs) [44,45]. Additionally, other peptide sequences have been immobilized on different scaffolds, such as Tyr-Ile-Gly-Ser-Arg (YIGSR) and Ile-Lys-Val-Ala-Val (IKVAV) in laminin [46], as well as Arg-Glu-Asp-Val (REDV) and Leu-Asp-Val (LDV) in FN [47]. Peptide fragments from collagen have been used for surface modification in numerous studies. For example, Bhatnagar and colleagues identified a cell-binding domain P15 (GTPGPQGIAGQRGVV) from type I collagen that supported ECM synthesis [48]. Similarly, another type I collagen peptide-GFOGER was used to functionalize surfaces and shown to support the expression of osteogenic genes and to induce matrix mineralization, similar to type I collagen [49,50]. Recently, Aziz and co-workers have developed a 3D matrix metalloproteinase (MMP)-sensitive hydrogel to promote osteocyte differentiation. These poly(ethylene glycol) hydrogels contained the peptide crosslink GCGPLG-LWARCG (MMP-sensitive peptide) and RGD to promote cell attachment. This hydrogel was capable of cell-mediated degradation and enhanced mineralized collagen matrix and osteocyte differentiation [51].

Collagen has been described to be extremely important as a template to create bone tissue; however, it is unknown which of the other components from bone matrix are essential to engineer new bone tissue. Recently, investigators have been focusing on using the native non-collagenous proteins from bone matrix, instead of collagen-binding motifs, to enhance the properties of the scaffolds, such as cell migration, proliferation and osteogenesis. Although great improvements in developing peptide-incorporated biomimetic materials have been achieved, the influence of the non-collagenous bone matrix proteins on osteogenic differentiation remains to be evaluated, even though they have been identified and described some decades ago [19]. Thus, in vitro and in vivo studies must be conducted to better understand the functions of the bone matrix constituents. In fact very few studies have used components of the organic bone matrix other than collagen to create bone substitutes. Johnson and colleagues used a composite alloimplant of human bone morphogenetic protein and autolyzed allogeneic bone containing a mixture of ECM proteins. In a clinical trial, bone union was possible in 24 of 25 cases identified as non-unions. However, the addition of BMPs as osteoinductive factors did not allow for any conclusions on the performance of the non-collagenous bone matrix proteins alone [52]. Sun and co-workers integrated non-collagenous proteins from bone ECM into gelatin scaffolds to form an artificial matrix that mimicked natural ECM while enhancing osteogenesis and mineralization [53].

In the next section, we will introduce and briefly summarize the several non-collagenous proteins present in bone ECM, as well as describe their key characteristics in context of bone tissue engineering (Table 1).

### 4.1. Proteoglycans

This class of molecules is characterized by the covalent attachment of long chain polysaccharides (glycosaminoglycans, GAGs) to core protein molecule. GAGs are composed of repeating carbohydrate units that are sulfated to varying degrees and include chondroitin sulfate (CS), dermatan sulfate (DS), keratan sulfate (KS), heparin sulfate (HS), and hyaluronan (HA, unsulfated) [31]. These proteins are commonly found in cartilage matrix. Moreover, since endochondral bone formation is mediated by a cartilage matrix, chondrogenic proteins can be incorporated into the initial matrix to promote bone formation [31,32]. Proteoglycans have been suggested to be responsible for matrix maintenance, organization and regulation of cartilage calcification, through interactions with the GAG chain of type IX and the type II collagen fibrils [62]. Proteoglycans and GAGs can inhibit hydroxyapatite formation and growth [63] and they can also chelate. Several proteoglycans have been identified in cartilage matrix and bone matrix, such as the large proteoglycans, aggrecan and versican, and small leucine-rich repeat proteoglycans, such as decorin and biglycan [64].

For tissue engineering applications, synthetic peptido-GAGs have shown promising results for biomedical applications [65]. These peptido-GAGs could replicate many biological functions of decorin, modulating fibril formation and stiffness of the new tissue and promoting cellular adhesion [66,67]. Also, aggrecan peptido-GAG have been used to enhance the properties of some scaffolds for cartilage regenerative applications [68].

Moreover, few studies have incorporated collagen and GAGs within scaffolds [68,69], providing a suitable 3D environment for inducing osteogenic differentiation of MSCs [70,71] and to enhance osteoblast activity [72]. In fact, incorporation of GAGs within collagen fibrils improved cell proliferation [73]. Moreover, the addition of GAGs reduced the degradation rate of the scaffold and improved the structural stability of the collagen/GAG matrix [73,74]. Interestingly, these scaffolds have demonstrated favorable results for bone tissue engineering applications, even without adding stem cells or growth factors to the system [70]. Therefore, approaches to mimic the native structure and composition of bone tissue with novel scaffolds comprising collagen, GAGs and calcium phosphate crystals have been successful [75,76].

The glycosaminoglycan CS has also been used for bone tissue engineering applications as CS supports osteogenic differentiation of MSCs and increases the regeneration ability of injured bone. A CS-bioglass composite encapsulating MSCs was reported to induce bone regeneration in vivo when incorporated with BMPs [77]. Also, a CS-collagen biomaterial, fabricated as a BMP delivery system, showed high biocompatibility and osteogenic stimulation [78]. Hyaluronic acid has been used in several medical fields. This GAG is also used as a carrier for regenerative growth factors [79]. Proteoglycans can be further divided into large proteoglycans (aggecan and versican) and small leucine-rich repeat proteoglycans (decorin and biglycan) (Table 1 and Table 2).

#### 4.1.1. Large Proteoglycans

Aggrecan and Versican are two large CS-proteoglycans that are found in bone matrix and can bind to HA, forming large aggregates.

##### Aggrecan

Aggrecan has been suggested to play an important role in skeletal development, having a molecular weight of approximately 205 kDa. First studies revealed that mice with a mutation of aggrecan gene presented cartilage matrix deficiency, perinatal lethal dwarfism and craniofacial abnormalities [80]. Since the amount of aggrecan present in bone is much lower than in cartilage tissue, it is not completely understood if its presence in bone represents residual calcified cartilage. Although aggrecan has been reported to have an important role in preventing cartilage calcification, its function in bone remains unknown.

##### Versican

Versican is another CS proteoglycan that is found in relatively lower levels compared to aggrecan in cartilage and bone. It has a molecular weight of approximately 360 kDa and is reported to be expressed during osteogenesis and in bone development [81]. Although versican stimulates chondrocyte proliferation [82], its function in cartilage and bone are still unknown. Potentially, it can serve as a bridge between cells and the ECM, allowing for cell binding to HA [83].

#### 4.1.2. Small Leucine-Rich Repeat Proteoglycans

Small leucine-rich repeat proteoglycans are another family of proteoglycans with a protein core and leucine-rich repeat sequence. In cartilage and bone, several members of this family, such as decorin and biglycan, are present. They exhibit different patterns of expression and tissue localization, which might be indicative of different functions.

##### Decorin

Decorin has an apparent molecular weight of approximately 130 kDa and it has been shown to bind to and regulate the fibrillogenesis of type I, II, and VI collagens and collagen-matrix interactions [90]. In bone, decorin may function as regulator of collagen fibril diameter and fibril orientation and can prevent premature osteoid calcification due to its low affinity for calcium [84,90], in contrast to a high affinity to type I collagen. Moreover, studies suggest a role of decorin in matrix mineralization, since proteoglycans with low molecular weight are present in type I collagen fibrils but then disappear when mineralization occurs [85]. Studies with decorin-knockout mice showed skin laxity and fragility without any visible changes in bone phenotype. However teeth from decorin-knockout mice showed alteration in matrix properties, presenting a hypomineralized dentin [86].

##### Biglycan

Biglycan is another small proteoglycan present in both cartilage and bone with a molecular weight of approximately 45 kDa. Although its functions remain to be investigated, biglycan demonstrates different effects in solution depending on its concentration. Low concentrations promote apatite formation whereas in higher concentration biglycan inhibits the growth and proliferation of mineral crystals [84].

The biglycan-knockout mice present reduced skeletal growth with short femora and decreased bone mass [87]. Moreover, decorin and biglycan-double knockout mice have additive deficiency in dermis and synergistic effects in bone. Ultrastructural analysis of these mice reveals loss of fibril geometry [88]. The mineral within these bones has increased crystal size compared to wild-type controls [89]. However, the low amount of biglycan in bone matrix as well as its absence within bone collagen fibrils suggest that its principal function may not be directly related to mineralization.

### 4.2. Glycoproteins

This class of proteins is characterized by the covalent linkage of sugar moieties attached via asparaginyl or serinyl residues [32]. These glycoproteins may be further modified by post-translational sulfation and phosphorylation (Table 1 and Table 3). Glycoproteins derived from bone matrix have been reported to enhance cell binding, being recently used in association with a variety of different scaffolds for tissue engineering applications.

#### 4.2.1. Alkaline Phosphatase

Although ALP is not typically thought of as a matrix protein, several studies demonstrated that ALP can be released from the surface of osteogenic cells or in a membrane-bound form (matrix vesicles) [91,92].

Developmental studies, in vivo and in vitro, have suggested an important role of ALP in mineralization, since its expression precedes mineralization and it is maintained during early stages of hydroxyapatite deposition [93]. Moreover, it is reported that hypophosphatasia is characterized by mutations in ALP gene and results in improper mineral deposition. Indeed, mice with null mutations for the tissue-nonspecific ALP showed increased osteoid and defective growth plate development, reinforcing the importance of ALP in mineralization [94]. Furthermore, it was observed that cells that normally do not mineralize promote formation of a mineralized matrix when transfected with the ALP gene [95].

For bone tissue engineering applications, ALP has been immobilized on microporous nanofibrous fibrin scaffolds [54]. These scaffolds are nontoxic, biodegradable, and support cell proliferation and osteogenic differentiation in vitro. Furthermore, the immobilized ALP fibrin scaffolds were shown to support bone formation in a mouse calvarial defect model. ALP has also been coated onto titanium scaffolds exhibiting increased hydroxyapatite formation while enhancing the bioactivity of titanium scaffolds [153]. Additionally, ALP immobilized on collagen and alginate scaffolds have been shown to be a good candidate for improving osteogenesis [154,155].

In a different study, ALP was successfully incorporated into halloysite (HAL) nanotubes. Immobilized ALP effectively induced biomineralization processes, thus, ALP-HAL nanocomposite material may be an attractive bioactive scaffold for bone regeneration [156].

#### 4.2.2. Osteonectin

Osteonectin (ON), a secreted phosphoprotein acidic and rich in cysteine (SPARC), was the first matrix protein to be isolated from bone with a molecular weight approximately between 35–45 kDa [96]. Although it is synthesized by osteoblasts, it can also be synthesized by fibroblasts from skin, tendon, sclera, and periodontal ligaments; however, most of ON found in circulation is derived from platelets [97].

ON binds to type I, type III, and type V collagen, thrombospondin and to hydroxyapatite through high-affinity calcium-binding sites [98,99,100,101]. Its affinity for calcium and phosphate ions may suggest that ON can promote mineral deposition; however, ON accumulates only within mineralized matrix, suggesting that it is not involved in the induction step of mineralization, but may have an important function in regulating growth and proliferation of mineral crystals. ON-deficient mice present poor bone condition and develop osteopenia with significant loss of trabecular bone associated with a decreased rate in bone formation [101,102]. These results reinforce that ON might support bone remodeling and maintenance of bone mass [101]. In vitro studies use both intact molecule and peptides derived from different regions of ON. Results suggest that ON is an important regulator of cell–matrix interactions [103]. However, many of these properties have not been evaluated in osteoblast cell cultures.

For bone tissue engineering perspective, few studies have incorporated ON into scaffolds to regulate the mineralization process [157]. A nano-hydroxyapatite/collagen/ON complex was developed to mimic the hierarchical structure of native bone from nanoscale to microscale, which surpasses the limitation of mineralized pure collagen synthesized in vitro [158]. Interestingly, the formation of the mineralized collagen nanofibers was influenced by the presence of ON [159]. Glycine–histidine–lysine (GHK) peptide, a fragment of ON, has been studied for tissue engineering applications. GHK was incorporated in alginate hydrogels and found to promote production of vascular endothelial growth factor (VEGF) and basic fibroblast growth factor (bFGF) from MSCs, increasing MSC proliferation [160]. Recently, biomaterial functionalization with GHK has been suggested to be beneficial for bone tissue engineering applications [55]. Klontzas and colleagues developed novel oxidized alginate hydrogels with the GHK peptide and demonstrated enhanced MSC osteogenic differentiation, suggesting a mechanism of GHK action related to integrin β1 and mediated by integrin linked kinase [55].

#### 4.2.3. Tetranectin

Tetranectin has an approximate molecular weight of 21 kDa [104]. This glycoprotein is expressed by osteoblasts undergoing mineralization and is also found in tumors undergoing mineralization [104]. To date, the exact function of tetranectin in bone metabolism is not known; however, this protein might be involved in matrix mineralization. Overexpression of tetranectin by tumor cells causes an increase in ECM mineralization upon implantation into nude mice [104]. Tetranectin-deficient mice show delayed fracture healing, indicating that tetranectin may have a role during the early stage of the fracture healing process [105]. Additionally, tetranectin-knockout mouse present a phenotype with severe spinal deformities [106].

Although tetranectin has been thought to have a potential role in mineralization during osteogenesis in vivo and in vitro [104], this protein has not been applied to engineer bone. Although not within the scope of this review, tetranectins have been engineered to target proteins on tumor cells with greater specificity than on normal cells [161].

#### 4.2.4. RGD-Containing Glycoproteins

Bone ECM also contains some glycoproteins with amino acid sequence RGD. These RGD sequences can be recognized by cell surface receptors and promote attachment between ECM and cells [162]. The receptors on the cell surface are integrins formed by one α subunit and one β subunit. Each subunit has a cytoplasmic extension that is associated with intracellular signaling pathways, a transmembrane domain and an extracellular domain [162]. The extracellular domains of the α and β subunits configure a binding pocket that recognizes the RGD sequences in the ECM proteins and mediates the cell-matrix interactions [162]. In bone matrix, some RGD-containing proteins include thrombospondin, FN, vitronectin (VN) and a family of small integrin-binding ligand, N-linked glycoproteins (SIBLINGs). The SIBLINGs have been identified by a cluster of genes including OPN, BSP, dentin matrix protein 1 (DMP-1) and dentin sialophosphoprotein (DSPP) [163].

RGD sequence has been reported to provide support for cell adhesion for bone tissue engineering; however, the RGD sequence may also enhance other cellular processes that promote mineralization [58]. Because RGD peptides interact with multiple cell types, there is a great need to identify peptide sequences that elicit more specific responses from particular cell types [58].

##### Thrombospondin(s)

Thrombospondin is a glycoprotein with a molecular weight of approximately 450 kDa [107]. Unlike other glycoproteins present in the bone ECM, thrombospondins are less abundant in mineralized bone matrix, but these proteins can be found in several connective tissues. Although their role in bone is still not known, thrombospondins have been suggested to be important in bone development and remodeling, especially in collagen fibrillogenesis and ECM organization [108,109,110]. Moreover, thrombospondins can bind to several matrix proteins and cell surface proteins [31]. Studies with mice that lack thrombospondin (TSP-2 null) present disordered collagen in their soft tissues [111] with increased cortical bone thickness and density [108].

Thrombospondin 1 (TSP-1) has been shown to influence neovascularization when incorporated in porous polyethylene implants. These findings represent a new promising approach for enhancing angiogenic properties of biomaterials for bone tissue engineering applications [164].

##### Fibronectin

Fibronectin (FN) is one of the most abundant ECM proteins in bone with a molecular weight of approximately 400 kDa. Since it is produced by all connective tissue cells, this protein can be found in all ECM within the body [112]. Evidence suggests that FN plays an important role during bone development. It accumulates in mineralized matrix at an early stage of bone formation [113] and is highly up-regulated by osteoblasts. In vivo studies showed that FN is an essential component for the development of the connective tissue, since the elimination of the FN gene in transgenic animals is lethal in utero, due to the lack of formation of connective tissues [114].

Interestingly, the attachment of osteogenic cells to FN, in vitro, is independent of RGD-mechanism [113]. Moreover, for cell-matrix interactions mediated by FN, α_4_β_1_ binding may play a role in the maturation sequence of cells in the osteogenic lineage [32].

Recently, some studies have been focused on improving scaffolds’ ability to target and recruit stem cells in order to accelerate cell adhesion—a promising tool for bone tissue engineering. To this end, FN has been used for surface modification by coating this protein on bioactive scaffolds to promote cell adhesion and proliferation [165]. Although ECM’s affinity for cell adhesion has been well reported, the optimal ECM coating for osteogenic differentiation remains unclear. Therefore, studies have combined more than one ECM protein to coat the biomaterial’s surface with a goal to enhance cell adhesion and differentiation for developing functional constructs for clinical skeletal regeneration. Moreover, by using a robotic-dispensing technique, it is possible to generate FN-immobilized nanobioactive glass (nBG)/polycaprolactone (PCL) (FN-nBG/PCL) scaffolds with an open pore architecture. With the addition of these cell-adhesive motifs onto the surface of the scaffold, cellular adhesion and differentiation processes can be accelerated [56]. Recent strategies involve the combination of biomaterials with ECM proteins or growth factors and hydroxyapatite as a promising application for bone reconstruction [166]. For example, Toupkanlou and colleagues incorporated nanohydroxyapatite in electrospun nanofibrous polycaprolactone (PCL) scaffolds coated with FN, demonstrating the synergistic effect of FN and hydroxyapatite on enhancing calcium deposition, collagen synthesis and early ALP activity and upregulation of osteogenic specific genes in vitro and in vivo [166]. Similarly, Lee and co-workers constructed a novel osteoinductive FN matrix fusion protein (oFN) containing FN_III9_ and FN_III10_ modules, the key cell-binding domain of FN, and an osteoinductive sequence from BMP-2 [165]. The engineered oFN matrix fusion protein resulted in more effective bone regeneration via promotion of cellular adhesion and differentiation. These studies show that the design of fusion proteins could represent a highly relevant approach for bone tissue engineering.

Recently, Trujillo and colleagues engineered 3D hydrogels with full-length fibronectin that sequesters growth factors, such as VEGF and BMP-2, to enhance angiogenesis and bone regeneration. The physical properties of this hydrogel can be tuned to mimic the properties of the native ECM, providing a novel 3D environment with the potential to recruit and retain growth factors promoting bone regeneration and vascularization in vivo [167].

Recent advances in bone tissue engineering have varied ECM properties by incorporating decellularized ECM from tissues or cells onto different scaffolds. For example, silk fibroin scaffolds have been coated with decellularized pulp/collagen/FN and the modified scaffold has demonstrated promising results for bone tissue engineering applications [168].

##### Vitronectin

Vitronectin (VN) has a molecular weight of approximately 70 kDa and it is generally found in matrices containing fibrillary collagens. In vitro, VN is produced by osteoblasts [113]. This protein plays an important role in cell adhesion. Osteogenic cells, including osteoclasts, attach very strongly to VN, mainly via the receptor integrin α_ν_β_3_ [113]. Interestingly, VN increases its concentration in the unmineralized osteoid prior to mineral deposition [115]. This evidence indicates that VN can have an important role in preparing the matrix for mineralization. In vivo studies demonstrated that mice that lack VN gene presented a thrombolytic phenotype; however, skeletal defects were not observed in these mice [116].

Similar to FN, VN has been used to coat bioactive scaffolds. Cacchioli and colleagues investigated the effect of VN peptide conjugated onto titanium surfaces and showed an increased cell apposition rate, higher ratio of mineralized surface to bone surface and more extended bone-to-implant contact in in vivo models [57].

##### Osteopontin

OPN is part of a family of five integrin-binding glycophosphoproteins (SIBLINGs). Besides OPN, this family comprises BSP, DMP-1, DSPP and matrix extracellular phosphoglycoprotein. The genes coding for members of the SIBLING protein family are similarly organized. SIBLINGs interact with cell surface receptors, such as integrins, mainly through a RGD sequence and function as modulators of cell adhesion as well as autocrine and paracrine soluble factors [169].

All of these proteins undergo similar post-translational modifications such as phosphorylation and glycosylation, the extent of which is crucial in determining their function [170].

OPN, also called secreted phosphoprotein (SPP), is an acidic glycoprotein that consists of about 300 amino acids, with a molecular weight of 34 kDa [117]. OPN was first identified in bone matrix acting as the bridge between cells and hydroxyapatite in bone ECM [118]. However OPN can also be detected in other tissues and plasma, such as dentin, cartilage, kidney and vascular tissues. In these tissues, OPN mediate communication between cells, suggesting that OPN could act both as a structural molecule and as a cytokine [119,120]. In bone, OPN is produced by osteoblasts during the pre-mineralization and at late stages of osteoblastic maturation [117]. OPN binds to α_ν_β_1_, α_ν_β_3_, α_ν_β_5_, α_4_β_1_, α_5_β_1_, and α_9_β_1_ integrins [118,121], through their RGD domain. Additionally, OPN can also present a RGD-independent mechanism, in which OPN may engage CD44 [117,118]. However, details of the interaction of CD44 with OPN remain to be studied. The presence of additional cell receptors, the various isoforms of CD44 and variable post-translational modifications (phosphorylation and glycosylation) of OPN are all complicating factors. Moreover, α_ν_β_3_ integrin is considered to be responsible for major signals in response to the binding of OPN [122]. OPN has been proposed to regulate many physiological processes such as collagen organization, cell adhesion, cell viability, cell migration, angiogenesis, and calcification [117,123]. OPN interacts with several molecules present in the bone matrix. OPN is known to bind covalently to FN via transglutamination, and transglutamination of OPN increases its binding to collagen [124]. Ritter and colleagues demonstrated that OPN specifically associates with OC, forming stable complexes between OPN and OC [27]. In fact, the mechanisms responsible for bone formation and remodeling likely involve the association of bone matrix proteins into specific complexes that helps the organization of the matrix [27]. Moreover, the phosphorylation of OPN has shown significant effect on crystal growth [125], regulating bone crystal size. OPN has a high affinity to calcium, therefore it has been suggested to modulate the nucleation of calcium phosphate during mineralization [126]. Besides the RGD sequence (amino acid sequence Arg159-Asp159), OPN also contains an aspartate domain at its N-terminal composed of amino acid sequence Asp86-Asp89 and a calcium binding domain (amino acid sequence Asp216-Ser228) with high negative charge motifs that might be responsible for the binding of OPN to bone mineral [126,127]. However, initial studies on OPN-deficient mouse failed to indicate the presence of any major defect in mineralization [127]. Possibly, the role of OPN in bone mineralization is compensated for by other regulatory systems for mineralization or by other non-collagenous bone ECM proteins.

Further studies of genetically OPN-knockout mice showed that these mice presented larger crystal size and an increased mineral content [128], suggesting the inhibitory role of OPN in hydroxyapatite formation and growth [128,129]. Analysis of the OPN^−/−^ mouse has also demonstrated that OPN is important for the function and activity of osteoclasts, specifically in osteoclast attachment and bone resorption [127,130]. Studies of ectopic bone implantation demonstrated that bone from OPN-deficient mice implanted into OPN-deficient mice exhibited significantly less resorption and lower number of osteoclasts attached to the surface of the bone compared to wild-type bone implanted intramuscularly in the back of the wild-type mice (5% vs. 25%) [130]. However, it is not known whether OPN promotes bone resorption by stimulating angiogenesis or by stimulating bone resorption via signaling through the bone matrix. Asou and colleagues demonstrated a relationship between OPN and bone resorption associated with vascularization [130], since the number of CD34^+^ vessels near the bone implanted in OPN-deficient mice were reduced compared to wild-type, suggesting that OPN deficiency may lead to a reduction in neovascularization of ectopically implanted bones, and a consequent reduction in the number of osteoclasts and bone resorption efficiency. It is also possible that OPN may promote the survival of endothelial cells on bone matrix, facilitating the vascularization of bone tissue. However, further investigation is required to elucidate the molecular mechanisms of OPN action in mediating responses to inflammation, mechanical stress, angiogenesis, and accelerated bone resorption.

Regarding bone tissue engineering, OPN plays an important role in cell adhesion, remodeling and osseointegration at the biomaterial/tissue interface that is commonly found surrounding mineralized tissues [171]. More specifically, OPN is responsible for the recruitment of osteoblasts during the early stage of bone formation [128]. Somerman and co-workers showed that the attachment of osteoblasts to OPN was dose-dependent and was mediated by a conservative RGD peptide sequence [172]. Some peptides were already derived from OPN and have been shown to bind to collagen and promote biomineralization [117,171]. Work by Shin and colleagues showed that oligo(poly(ethylene glycol)) fumarate hydrogels modified with OPN-derived peptide influenced osteoblast proliferation and migration, demonstrating a high potential of these biomimetic materials [53,58]. OPN can also interact with multiple cell surface receptors and play an active role in many physiological processes like wound healing, bone turnover, inflammation and angiogenesis [117]. Moreover, we demonstrated that OPN dose-dependently increased the proliferation of MSCs, as well as improved the angiogenic properties of human umbilical vein endothelial cells by increasing the capillary-like tube formation in vitro [173]. For bone tissue engineering applications, a new biomimetic strategy to rapidly form mineralized bone tissue and secure a sustained bone formation response by MSCs was then developed by incorporating OPN and OC in type I collagen hydrogels [174]. He and colleagues described a hydrogel that combined OPN, BMP-2 and RGD sequence to provide a favorable microenvironment for osteogenic and vasculogenic differentiation of MSCs [175]. Lee and colleagues showed that a collagen-binding motif (GLRSKSKKFRRPDIQYPDATDEDITSHM) found in OPN could specifically bind to collagen without chemical conjugation and also demonstrated its capacity to promote hydroxyapatite formation in vitro and in vivo [176]. OPN has been reported to induce bone formation by inhibiting osteoclast resorption and, more importantly, by increasing angiogenesis [118]. In fact, it has been reported that the peptide SVVYGLR, corresponding to amino acids residues 162–168 of OPN, induces vasculogenic differentiation of MSCs [59,177,178]. This peptide induces tube formation by progenitor endothelial cells in 3D collagen gels with as much potency as VEGF [177]. Hamada and co-workers developed CO_3_ apatite-collagen sponges containing the SVVYGLR motif and implemented the strategy to repair defect created in rat tibia. The scaffold promoted angiogenesis inside the graft, highlighting the importance of OPN incorporation into biomaterials for bone tissue engineering applications [59]. Zhu and colleagues developed 3D printed scaffolds composed by OPN sequenced peptide SVVYGLR grafted into mesoporous calcium silicate. Interestingly, the peptide motifs can be accessed on the surface of the scaffolds and can be released by the scaffold. In vitro and in vivo studies revealed enhanced angiogenic and osteogenic properties of these scaffolds [179].

Recently, plant-derived recombinant human OPN (p-rhOPN) has been conjugated to different scaffolds for bone tissue engineering applications [180,181]. Klinthoopthamrong and co-workers developed an active non-resorbable guided tissue regeneration membrane from bacterial cellulose combined with p-rhOPN [181]. These p-rhOPN membranes elicited biological functions leading to the enhancement of osteogenic differentiation of human periodontal ligament stem cells [181].

##### Bone Sialoprotein

In addition to OPN, BSP is another major SIBLING that accumulates in cement lines and in spaces between mineralized collagen fibrils [131]. BSP has an apparent molecular weight of approximately 75 kDa and its expression is exclusively located to the mineralized tissues, such as bone, dentin, cementum and certain regions of hypertrophic chondrocytes [131,132]. In bone, BSP is highly expressed by osteoblasts, osteoclasts, osteocytes and chondrocytes [132]. In fact, BSP expression indicates a late stage of osteogenic differentiation and an early stage of matrix mineralization. In addition, BSP has a high affinity for calcium, indicating that BSP is important for matrix mineralization [169]. In vitro, BSP might act as a hydroxyapatite nucleator [133]. It has been shown that a concentration as little as 9 nM BSP is sufficient to nucleate hydroxyapatite. The overexpression of BSP in osteoblasts has been shown to enhance mineralization [134]. Similarly, osteoblasts grown in the presence of an anti-BSP antibody exhibit reduced mineralization [135]. Interestingly, a cooperative relationship between BSP and collagen has been reported to increase nucleation potency when both proteins are linked [136]. In vitro assays also confirmed the role of BSP in mediating cell attachment, most likely through interaction with the α_ν_β_3_ receptor, facilitating the in vitro attachment of fibroblasts, osteoblasts and osteoclasts [169]. Moreover, BSP increases osteoclasts formation and bone resorption [137]. First in vivo studies demonstrated that BSP-deficient mouse does not exhibit an altered skeletal phenotype, possibly due to compensation of BSP function by other SIBLING proteins, revealing no differences in mineral crystal characteristics relative to controls [138]. However, more recent studies with BSP-deficient mouse show that it displays shorter, hypomineralized bones with associated higher trabecular bone mass and low bone turnover [137].

Many groups have explored the use of BSP to repair bone defects by enhancing their osteoinductive capacity [137,138]. In fact, BSP implants are attractive candidates for bone applications since BSP plays an important role in osteogenic differentiation by binding to type I collagen and to α_ν_β_3_ and α_ν_β_5_ integrins, and by mediating cell signaling and differentiation [134]. Moreover, BSP has been reported to enhance osteogenic differentiation of MSCs cultured on type I collagen [135]. Due to its high affinity for collagen, BSP combined with collagen facilitates cell migration, attachment, proliferation and differentiation through RGD and non-RGD binding of integrins [182]. Instead of using the whole protein, some investigators have been exploring the properties of the amino acids sequence 35–62 of rat BSP, corresponding to the collagen-binding peptide derived from BSP [182]. In vivo studies demonstrated that hydroxyapatite implants containing BSP-derived collagen-binding peptide implanted into rabbit calvarial defects promote new bone formation within two weeks after implantation as compared to untreated or hydroxyapatite scaffolds alone [183]. Several groups have also shown that the presence of BSP in some collagen implants, in vivo, stimulates osteogenic differentiation and bone repair [182,183,184] by up-regulating the expression of osteogenic genes associated with early differentiation. Interestingly, implanted BSP-collagen scaffolds, by day 7 after surgery, promote cell proliferation, matrix mineralization, and vascular invasion thereby extending bone formation into the central regions of the BSP-collagen implants. In contrast, when using only collagen scaffolds, the central regions of the implant are not affected [182]. Thus, defects where BSP-collagen scaffolds were implanted, presented new bone formation and remodeling in the whole areas of the defect, whereas defects that were implanted with collagen alone only demonstrated new bone formation in the areas near the host bone [182]. Moreover, another group has shown that functionalized silk-BSP scaffold enhanced osteogenesis, inducing mineralization and osteogenic differentiation of human MSCs, when compared to cells cultured in the presence of silk alone [185]. Furthermore, using synthetic polymers, Chan and colleagues have shown that some biomaterials that were initially thought to be used for cartilage tissue engineering applications can also be used in bone tissue engineering field [186]. Therefore, they modified polycaprolactone/poly(2-hydroxyethyl methacrylate) (PCL/pHEMA) surfaces with BSP, demonstrating an enhancement in cell adhesion, likely mediated through cell-surface receptors for RGD sequences. These enhanced cell-surface interactions found on BSP surfaces not only promote regeneration of bone, but also assist other important cellular events, such as proliferation, differentiation and matrix synthesis. Furthermore, other investigations have focused on the BSP-RGD peptide, instead of the entire protein. For example, Rezania and colleagues have covalently grafted BSP-RGD peptide from rat/mouse BSP onto quartz surfaces and shown that this peptide promotes ECM mineralization [187]. In a different study, Drevelle and co-workers used PCL films functionalized with BSP-RGD peptide and demonstrated an enhancement of cell spreading using MC3T3-E1 mouse pre-osteoblasts with an improvement of their responsiveness to recombinant human BMP-2 [188]. More recently, this BSP-RGD peptide has been reported to increase mineralization of human MSCs cultured on hydrogels, enhancing the expression of osteogenic gene markers [189]. Similarly, Rapuano used a fragment from human BSP corresponding to residues 278–293 and showed that BSP-coated plastics have better adhesion capacity, since more MC3T3-E1 cells were found attached to these surfaces [190]. In vivo studies evaluated BSP coating of 3D printed calcium phosphate scaffolds in a calvarial defect model in mice. Although histological analyses revealed that BSP-coated scaffolds had a better integration in the bone defect, no significant increase in bone formation was observed in BSP-coated scaffolds [60,61].

##### Dentin Matrix Proteins

Dentin matrix proteins (DMPs) are a group of non-collagenous proteins found in the ECM of dentin and bone, namely dentin matrix protein 1 (DMP-1), dentin phosphophoryn (DPP) or dentin matrix protein 2 (DMP-2), dentin sialoprotein (DSP), and dentin matrix protein 4 (DMP-4) [191]. Over the years, these DMPs have been shown to play multiple roles, such as in cell attachment, proliferation, differentiation, and matrix mineralization.

DMP-1 was first isolated from dentin, however, it can also be found in bone [139]. In bone, DMP-1 is expressed specifically in mineralized tissues by hypertrophic chondrocytes, osteoblasts, and osteocytes [140]. DMP-1 is a highly phosphorylated protein with a strong affinity for calcium. DMP-1 has been reported to influence mineralization, facilitating nucleation of hydroxyapatite crystals [141]. In fact, MC3T3 cells overexpressing DMP-1 promoted ECM mineralization [142]. Recombinant human DMP-1 has been shown to induce the osteogenic differentiation of human periodontal ligament cells [143].

Moreover, when phosphorylated, full-length DMP-1 inhibits the formation and growth of hydroxyapatite; however, when dephosphorylated, DMP-1 acts as a nucleator of hydroxyapatite formation [144]. Additionally, DMP-1 can bind specifically to the N-telopeptide region of type I collagen and, interestingly, nucleation of hydroxyapatite was exclusively found in regions where DMP-1 is bound to type I collagen [145]. DMP-1 has been reported to play a specific role in angiogenesis [146]. DMP-1-knockout mouse has further confirmed the potential role of DMP-1 in bone mineralization, since these mice have significant lower mineral content when compared to wild-type control mice [147,148].

Regarding tissue engineering, dentin has been explored to engineer the tooth structure. Compared to enamel, dentin is less mineralized and more elastic. It contains, approximately, 70% hydroxyapatite [192]. In fact, ECM proteins of human dentin are known to be necessary for dentinogenesis [193]. Several studies have attempted to create scaffolds that mimic the structure of natural dentin aiming to regenerate dentin [194]. Indeed, some reports have already demonstrated that treated dentin matrix could induce precursor cells to differentiate [192]. Dentin matrix has been fabricated to provide a natural, biocompatible scaffold, giving the appropriate microenvironment to induce complete human dentin tissue regeneration in vivo following implantation.

Current efforts are directed to study the role of the dentin matrix, specifically in tissue regeneration [195,196]. Since this is a new field in tissue engineering, there are a handful of studies incorporating specific dentin proteins/peptides into scaffolds for bone tissue engineering. However preliminary reports show that DMP-1 plays multifunctional roles and is potentially very attractive for tissue engineering applications [191]. To this end, Alsanea and colleagues demonstrated that dental pulp stem cells incorporated within a collagen scaffold in the presence of DMP-1 can differentiate into odontoblast-like cells, secreting a highly vascularized collagenous matrix [196]. These findings could be successfully applied to address clinical problems such as endodontic perforations, where the perforation site could be repaired using collagen scaffolds combined with dental pulp stem cells and DMP-1 signaling molecule.

Although DMP-1 was originally discovered from dentin matrix, it was also found in bone matrix and applied for bone tissue regeneration. Indeed, DMP-1-derived peptides have been shown to induce transformation of amorphous calcium phosphate to crystalline hydroxyapatite, demonstrating that this signaling molecule incorporated into a biomimetic scaffold could enhance nucleation of crystalline hydroxyapatite, generating high quality engineered tissues capable of withstanding the mechanical loading that bones are normally subjected to [197].

Using important domains of DMP-1, several peptides have been synthesized for incorporation into scaffolds designed for tissue engineering applications [191]. Besides the nucleating motifs of DMP-1, synthetic peptides that also contain type I collagen binding domain were generated for repair of carious dentin [191]. Thus, it may be possible to enhance the link between DMP-1 and collagen scaffolds and take advantage of the DMP-1 nucleating domain in order to facilitate calcium binding and mineralization. Recently, DMP-1 has been shown to stimulate osteogenic differentiation of MSCs [191]. Therefore, DMP-1 could be further explored in bone tissue engineering applications to enhance osteogenesis, by incorporating the whole protein or the nucleating domain of DMP-1. However, further studies are required to understand the potential role of DMP-1 as a signaling molecule for bone tissue engineering applications.

##### Dentin Sialophosphoprotein

DSPP is expressed in dentin, bone, cementum, and non-mineralized tissues including the lung and kidney [149]. As a single gene, an intact protein has not been isolated. However, two DSPP products, DSP and DPP, are co-expressed by odontoblasts and pre-ameloblasts at the same time as predentin is secreted [149]. Only DPP has been reported to regulate type I collagen fibrillogenesis [150] and acts as an effective nucleator for hydroxyapatite formation at lower concentrations and as an inhibitor at higher concentrations [151]. In contrast, DSP is not an effective modulator of in vitro mineralization. Some studies have shown that, when incubated with simulated body fluid solution, type I collagen scaffolds containing DPP are able to produce mineralized nodules similar to those found in bone [152], demonstrating the potential of DPP in bone tissue engineering applications for enhancing nucleation and growth of hydroxyapatite. Regarding DSP, this protein has not yet been used in bone tissue engineering applications, since its main function seems to be inhibition of mineralization [191]. In fact, negative regulators may be relevant for bone tissue engineering to allow for appropriate mineral deposition and for production of high quality mineral in cooperation with the positive regulators. Thus, DSP could be used with DPP to control the amount and quality of mineral produced for bone tissue engineering applications [191].

DSPP-knockout mice have shown decreased mineral content in dentin and bones [198,199], confirming importance of DSPP in mineralization. In humans, a mutation in the DSPP gene results in dentinogenesis imperfecta, characterized by dentin hypomineralization and significant tooth decay [200]. Some studies suggest that DSPP has roles not only in the initial mineralization of bone but also in the remodeling of the skeleton and therefore on bone turnover [201].

### 4.3. Gla-Containing Proteins

Bone contains several proteins that are post-translationally modified by vitamin K-dependent enzymes to form the amino acid, Gla [31] (Table 1 and Table 4). Osteocalcin is the major Gla-containing protein, playing an important role in mineralization of bone, whereas matrix Gla protein is known to be more involved in regulating the calcification of cartilage.

#### 4.3.1. Osteocalcin

Osteocalcin (OC), also known as bone gama carboxyglutamic acid-containing protein (BGLAP), is an approximately 5.8 kDa protein consisting of a single chain of 49–50 amino acids, being the most abundant non-collagenous protein in bone, comprising about 20% of the non-collagenous matrix proteins [202]. OC is secreted by osteoblasts and is present in dentine and calcified matrix. This protein has three glutamic acid residues at positions 17, 21, and 24 that bind calcium and it is vitamin K-dependent [203]. Before being released into the bone ECM, OC is carboxylated on its three glutamine acid residues within the osteoblasts, however, both the carboxylated and uncarboxylated forms of OC can be found in the circulation [203,204]. Its concentration in serum is closely linked to bone metabolism, being used clinically as a marker of osteoblast activity for the assessment of bone diseases [205]. During bone development, OC production is very low and does not reach maximal levels until late stages of mineralization [206]. Although its precise mechanism of action is unclear, OC is presumed to influence bone mineralization, in part through its ability to bind with high affinity to the mineral component of bone and due to its acidic character [207]. By binding hydroxyapatite, OC accelerates the nucleation of hydroxyapatite and plays an active role in the early stages of bone healing [208]. In addition to binding to hydroxyapatite, OC functions in cell signaling and in the recruitment of osteoclasts [209] and osteoblasts [210], which have active roles in bone resorption and formation, respectively. Subcutaneous implantation of bone particles that were 99% deficient in OC leads to poor bone resorption, suggesting that OC might function as a matrix signal in recruitment and differentiation of osteoclasts [211]. In vivo data showed that OC deficient mice present increased bone formation without impairing bone resorption [198]. Although the exact mechanism is still unknown, new studies have shown that the uncarboxylated form of OC may also act as a hormone regulating insulin secretion and glucose homeostasis [212,213]. However, its physiological role in mineralization remains uncertain.

An OC-derived scaffold was described by Rammelt and colleagues in which they investigated the addition of OC enhanced bone healing around hydroxyapatite/collagen composites in a rat tibia model. They demonstrated that OC activates both osteoclasts and osteoblasts during early bone formation [208]. A different study used recombinant human OC/FN_III9–10_ fusion protein to functionalize a collagen matrix for bone tissue engineering [214]. They demonstrated that OC/FN_III9–10_–functionalized collagen matrices are more effective in the osteogenic differentiation than non-treated collagen matrices or even FN_III9–10_–functionalized collagen matrices. These scaffolds could enhance cell adhesion, mostly by the FN domain but also were found to improve osteogenic differentiation. Regarding the effect of OC on angiogenesis, Cantatore and colleagues showed that OC exogenously applied to chick embryo chorioallantoic membrane stimulates angiogenesis and that the observed response was similar to that obtained with b-FGF [215]. Therefore, OC might be applied in bone regeneration to enhance angiogenesis in a defect site, improving the efficiency of bone healing. However, further assays are required to evaluate the angiogenic capacity of OC and its incorporation in scaffolds for bone healing applications.

#### 4.3.2. Matrix Gla Protein

Besides OC, matrix Gla protein (MGP) is the other major Gla-containing protein in the skeleton which was first isolated from bone [216] but can also be expressed in other soft tissues [207]. It has a molecular weight of approximately 15 kDa. MGP is known to be more abundant in cartilage than in bone [216]. In the skeleton, MGP expression appears early and remains at the same level at all stages of development [217]. There is evidence that MGP is an in vivo inhibitor of mineralization of cartilage. In vivo data shows that MGP-knockout mice die prematurely because of massive calcification of tracheal cartilage and blood vessels [218].

Although the precise mechanism through which MGP regulates bone metabolism is unknown, a recent study suggested that MGP may promote osteoblast proliferation, differentiation and mineralization via the Wnt/β-catenin signaling pathway [219].

### 4.4. Serum Proteins

Besides non-collagenous bone proteins, there are other proteins that are not synthesized in bone but can also be found in bone matrix, such as immunoglobulins, cytokines, chemokines, and growth factors. These proteins are mostly synthesized in the liver and in the hematopoietic tissue. Through circulation, these proteins accumulate in bone, especially due to their adsorption into bone ECM by hydroxyapatite [220]. Although these serum proteins are not produced locally, they may play an important role in bone metabolism. One example of a serum protein that can be found in bone ECM is albumin that is synthesized by the liver. In vitro studies show that albumin inhibits hydroxyapatite growth and influences hydroxyapatite formation [220].

### 4.5. Synergistic Biomimetic Strategies: Combination of ECM Proteins/Peptides to Elicit Bone Tissue Regeneration Responses

As it was mentioned before, depending on the end use, several combinations of functional domains of non-collagenous bone ECM proteins can be incorporated into scaffolds to elicit responses for bone tissue regeneration. Indeed, an additive or sometimes synergistic effect has been reported when combining more than one ECM protein/peptide. Most of the peptides incorporated into the scaffolds have integrin-binding RGD sequences to enhance cell binding to scaffolds; however, the addition of other ECM proteins, like BSP, OPN, or OC, has been shown to enhance mineralization, to accelerate bone healing, and to induce angiogenesis [173,174]. In that sense, a combination of non-collagenous bone ECM proteins might be an impressive strategy to improve the properties of a scaffold, giving it “ideal” cues to accelerate the process of bone healing. In fact, our group demonstrated that the loss of OC and OPN reduces stem cells self-renewal potential, osteogenic differentiation, and angiogenic potential [221]. Moreover, loss of OC and OPN compromises the ECM integrity and maturation, observed by an unexpected enhancement of glycosaminoglycans content that are associated with a more primitive skeletal connective tissue, and by a delay on the maturation of mineral species produced [221]. Thus, our group has developed a synergistic biomimetic strategy to develop matrices for bone tissue engineering applications by enhancing collagen matrices with OC and OPN, increasing MSCs proliferation and accelerating osteogenic differentiation [174].

### 4.6. Native ECM as a Biomaterial Source

Although progress has been made, new strategies are being developed by applying the native ECM as a biomaterial source to achieve the molecular complexity and organization of native tissue [222]. Indeed, native ECM can be obtained from allogeneic tissues (living donor/cadaver) or xenogenic tissues (animals) after cleaning and decellularization. In a graft obtained from decellularization of whole mature organ, the structure and tissue architecture are preserved [223] making it suitable for tissue engineering application [224]. However, there are some limitations of using native ECM as a graft. In fact, to reduce the risk of disease transmission, harsh decellularization treatments [225] are often required, leading to the loss of bioactive components in the ECM. Another limitation is the uncontrolled tissue variability that occurs due to the age, health or gender of the tissue donor [226].

Regarding bone tissue, bone ECM can be processed by treatment with acid [227] to generate demineralized bone matrix (DBM). DBM has a gel-like consistency that can be processed as powder or granules and, therefore, can be used as bone filling material, since it does not offer a structural support. Nevertheless, this material contains collagenous proteins and growth factors, such as BMPs, FGFs, and transforming growth factors. As mentioned before as a limitation of decellularized native ECM, DBM also has uncontrolled variability depending not only on the donor [228] but also on the sterilization method [229]. Therefore, it is quite impossible to predict and guarantee the osteoinductive properties of this material.

Decellularized ECM derived from cultured cells (cell-derived ECM) appears as an alternative to decellularized native tissue-derived ECM. Cell-derived ECM is composed by a complex and organized mixture of macromolecules that mimic the native tissue microenvironment and can be obtained by decellularization of in vitro cell cultures. Cell-derived ECM acts as a reservoir of multiple growth factors, such as factors involved in inflammation (i.e., monocyte chemoattractant protein 1, macrophage colony-stimulating factor, interleukin 8), angiogenesis (i.e., VEGF), and remodeling (i.e., matrix metalloproteinase 13, osteoprotegerin) [230].

Cell-derived ECM has greater ability for customization, in contrast to tissue-derived ECM. Cell-derived ECM allows for selecting the desired cell types and the culture system (2D vs. 3D, static vs. perfusion) to optimize ECM production. Furthermore, decellularized cell-derived ECM can be fabricated with specific properties by genetically modifying the cell sources to enhance the expression or sub-express some specific molecules [226]. Cell-derived ECM has been used also as coating by depositing molecules on the scaffolds surface to enhance their bioactivity and osteoinductive properties [231,232]. However, cell-derived ECM has also some limitations with respect to tissue-derived matrices. In general, cell-derived ECM has poorer mechanical properties making them unsuitable for some applications [231].

In vitro, cell-derived ECM has been shown to sustain cell expansion and to enhance MSCs osteogenic differentiation [232,233,234,235] and has been used for bone tissue engineering applications via incorporation into scaffolds and electrospun fibers [232,236]. In vivo, cell-derived ECM presents good vascularization [237] and is able to undergo remodeling onto an immature osteoid tissue [238].

## 5. Concluding Remarks and Future Perspective

Interest in the bone and dental tissue engineering field has seen tremendous growth over the years. Studies have focused on strategies that can ideally eliminate the drawbacks of current clinical approaches. Understanding bone/tooth structure, mechanics and tissue regeneration is also essential to successfully regenerate functional tissue. Much progress on coupling engineering with biology has been made over the last years but many challenges remain before bone tissue engineering becomes a true clinical reality.

Currently, great efforts are being made to find new solutions for designing and developing novel biomaterials that mimic ECM and recreate the appropriate bone/tooth niche, accelerating the healing process in a defect site. The potential of using biomimetic ECM peptides for bone tissue engineering applications has been investigated and confirmed by the increasing number of published works that report the effects of various ECM peptides on cells and bone regeneration. Furthermore, addition of peptides with different activities on the same scaffold might help in directing bone formation and healing more efficiently. However, better characterization of the different peptides is necessary in order to understand their synergistic effect. With this in mind, optimization of the administration strategy and control of the release rate of those peptides are extremely important and should be further investigated.

So far, very few studies using different non-collagenous proteins from bone matrix have been performed, although the increasing popularity of ECM peptides might help advance this field. Moreover, non-collagenous bone ECM proteins can be exploited as versatile tools for functionalizing scaffolds with osteoinductive signals to enhance cell adhesion, osteogenic differentiation and angiogenesis. Further studies need to be done to understand the mechanism of action of non-collagenous bone ECM proteins and how to use them to engineer new bone tissue for tissue engineering applications.

## Figures and Tables

**Table 1:** Non-collagenous protein-modified biomaterials for bone tissue engineering applications.

Non-Collagenous Protein	Modified Biomaterial	Outcomes	References
Alkaline Phosphatase	ALP-immobilized on microporous nanofibrous fibrin scaffolds by 1-ethyl-3-(3-dimethylaminopropyl)carbodiimide hydrochloride/N-hydroxysuccinimide (EDC/NHS) method.	Supported cell proliferation and osteogenic differentiation in vitro. In vivo, these scaffolds promoted bone formation.	[54]
Osteonectin	Oxidized alginate hydrogels with the GHK peptide, a fragment of osteonectin. The free aldehyde groups present in the oxidized alginate can form covalent bonds with molecules that contain amino groups, such as GHK (self-crosslinking).	Improved osteogenic differentiation of MSCs, demonstrated by enhanced gene expression, alkaline phosphatase activity and bone extracellular matrix deposition.	[55]
Fibronectin	Fibronectin-immobilized nanobioactive glass/polycaprolactone scaffolds by EDC/NHS treatment.	Improved cellular adhesion and proliferation.	[56]
Vitronectin	Vitronectin-derived peptide covalently grafted onto titanium scaffolds. Pretreated (oxidized and silanized) constructs were peptide-grafted by immersion overnight into a 1 mg/mL peptide solution.	The presence of the vitronectin-peptide bound to the titanium constructs improved the osteogenic activity immediately after implantation, accelerating bone ongrowth.	[57]
Osteopontin	Oligo(poly(ethylene glycol)) fumarate hydrogels modified with OPN-derived peptide. Peptides were coupled to acrylated-PEG by NHS treatment.	Improved osteoblast proliferation and migration.	[58]
Osteopontin	CO_3_ apatite-collagen sponges containing the SVVYGLR motif (amino acids residues 12–18 of OPN). CO_3_ apatite-collagen sponges were immersed in 10 ng/mL of SVVYGLR peptide solution.	In vivo studies presented improved angiogenesis.	[59]
Bone Sialoprotein	Bone sialoprotein coated 3D printed calcium phosphate scaffolds. 3D printed calcium phosphate scaffolds were coated with BSP via physisorption. Incubation was performed with different concentrations of BSP solution (50 and 200 µg/mL) under mechanical stirring at 8 °C.	Improved osteoblast viability and in vivo studies showed that BSP coated 3D printed calcium phosphate scaffolds promoted increased bone formation in comparison to uncoated scaffolds.	[60,61]

**Table 2:** Proteoglycans in Bone Matrix: Protein functions and in vivo studies.

Proteoglycans	In Vivo Studies	Functions	References
Aggrecan	Aggrecan deficient mice presented cartilage matrix deficiency and were characterized by perinatal lethal dwarfism and craniofacial abnormalities.	Can have an important role in preventing cartilage calcification.	[80]
Versican	Versican deficient mice have presented an early lethality.	Can have an important role in preventing cartilage calcification.	[81,82,83]
Decorin	Decorin-knockout mice showed skin laxity and fragility and their bones did not demonstrate any visible bone phenotype. However, their teeth sowed alteration in matrix properties, presenting a hypomineralized dentin.	Binds to collagen and can regulate fibril diameter and orientation. Can prevent premature osteoid calcification and regulate the collagen-matrix interactions.	[84,85,86,87,88]
Biglycan	The biglycan-knockout mice presented reduced skeletal growth, having shorter femora and decreased bone mass.	Binds to collagen and can regulate fibril diameter and orientation. Can prevent premature osteoid calcification and regulate the collagen-matrix interactions.	[84,86,87,88,89]

**Table 3:** Glycoproteins in Bone Matrix: Protein functions and in vivo studies.

Glycoproteins	In Vivo Studies	Functions	References
Alkaline Phosphatase	Mice with null mutations for the tissue –nonspecific alkaline phosphatase showed increased osteoid and defective growth plate development.	Possible role in mineralization. ALP can act as a potential Ca^2+^ carrier and hydrolyzes inhibitors of mineralization such as pyrophosphates.	[91,92,93,94,95]
Osteonectin	Osteonectin deficient mice have presented a poor bone status, developing osteopenia.	Can promote mineral deposition and regulate growth and proliferation of mineral crystals, supporting bone remodeling. May influence cell functions, binding to growth factors and through cell-matrix interactions.	[96,97,98,99,100,101,102,103]
Tetranectin	Tetranectin deficient mice have presented a delayed fracture healing.	Can regulate matrix mineralization, playing a role in tissue formation and remodeling.	[104,105,106]
Thrombospondin	Thrombospondin deficient mice presented disordered collagen in their soft tissues, increased cortical bone thickness and density and altered fibroblast attachment.	Role in cell attachment. It binds to several ECM proteins. Role in bone development and remodeling, collagen fibrillogenesis and ECM organization.	[107,108,109,110,111]
Fibronectin	Elimination of fibronectin gene in transgenic animals is lethal in utero, since connective tissues do not form.	Role in cell attachment. It binds to several matrix proteins and cell surface proteins, like collagen.	[112,113,114]
Vitronectin	Vitronectin deficient mice have been shown to have a thrombolytic phenotype, but skeletal defects were not apparent in these mice.	Role in cell attachment. It can bind to collagen.	[113,115,116]
Osteopontin	Osteopontin deficient mice presented larger crystal size and an increased mineral content.	Role in cell attachment. It binds with other molecules present in bone matrix. Can regulate mineralization by regulating the nucleation of mineral crystals. Can regulate bone resorption through osteoclasts attachment and migration. Play a specific role in angiogenesis.	[117,118,119,120,121,122,123,124,125,126,127,128,129,130]
Bone Sialoprotein	Bone sialoprotein deficient mice presented shorter, hypomineralized bones with higher trabecular bone mass and with lower bone formation rate.	Role in cell attachment and matrix mineralization induction. It acts as a hydroxyapatite nucleator since it has high affinity for calcium. Can have an important role in osteoclasts formation and bone resorption.	[131,132,133,134,135,136,137,138]
Dentin matrix protein-1	Dentin matrix protein-1 deficient mice have significantly lower mineral content when compared with their controls.	Role in cell attachment. It binds to collagen. If phosphorylated, may inhibit the formation and growth of hydroxyapatite, if dephosphorylated it facilitates nucleation of hydroxyapatite crystals, inducing mineralization. Can play a role in angiogenesis.	[139,140,141,142,143,144,145,146,147,148]
Dentin sialophosphoprotein	Dentin sialophosphoprotein deficient mice have shown decreased mineral content.	Can regulate type I collagen fibrillogenesis and acts as nucleator of hydroxyapatite formation at lower concentrations and inhibitor at higher concentrations.	[149,150,151,152]

**Table 4:** γ Carboxy glutamic acid-containing proteins in bone matrix: Protein functions and in vivo studies.

Gla-proteins.	In Vivo Studies	Functions	References
Osteocalcin	Osteocalcin deficient mice presented increased bone formation without impairing bone resorption.	Can influence bone mineralization. It has high affinity to calcium, accelerating nucleation of hydroxyapatite and playing an active role in the early stages of bone healing. Can regulate activity of osteoclasts and bone resorption. Acts as a hormone regulating insulin secretion and glucose homeostasis.	[202,203,204,205,206,207,208,209,210,211,212,213]
Matrix Gla Protein	Matrix Gla Protein deficient mice died prematurely due to massive calcification of their tracheal cartilage and blood vessels, indicating an important role in preventing mineralization.	Can function in cartilage metabolism inhibiting mineralization.	[214,215,216,217,218,219,220,221,222,223,224,225,226,227]

## Data Availability

The data presented in this study are available on request from the corresponding author.
